# Long-term follow-up on biological risk factors, adiposity, and cardiorespiratory fitness development in a physical education intervention: a natural experiment (CHAMPS-study DK)

**DOI:** 10.1186/s12889-018-5524-4

**Published:** 2018-05-09

**Authors:** Jakob Tarp, Eva Jespersen, Niels Christian Møller, Heidi Klakk, Barbara Wessner, Niels Wedderkopp, Anna Bugge

**Affiliations:** 1 Research Unit for Exercise Epidemiology, Centre of Research in Childhood Health, Department of Sports Science and Clinical Biomechanics, University of Southern Denmark, Campusvej 55, 5230 Odense M, Denmark; 2 Department of Rehabilitation, Odense University Hospital, Institute of Clinical Research, University of Southern Denmark, Odense, Denmark; 3 University College Lillebælt, Odense, Denmark; 4 Centre for Sport Science and University Sports, Department of Sports and Exercise Physiology, University of Vienna, Vienna, Austria; 5 Sports Medicine Clinic, The Orthopedic Department, Hospital of Lillebaelt Middelfart, Institute of Regional Health Research, University of Southern Denmark, Odense, Denmark

**Keywords:** Physical activity, School, Prevention, Cardiometabolic, Blood pressure, Insulin resistance, Lipids

## Abstract

**Background:**

Schools are a key setting for large-scale primordial non-communicable disease prevention in young people, but little data on sustainability of impacts on cardiometabolic risk markers is available.

**Methods:**

Six and a half year follow-up of a natural experiment. In 2008, six public schools in the municipality of Svendborg (Denmark) augmented their curricular physical education (intervention) and four matched schools served as controls. At long term follow up in 2015 *n* = 312 participants aged 5–11 years had complete data (33% of children providing necessary baseline data). The intervention, that consisted of a trebling of weekly physical education lessons and courses provided to physical education teachers, was provided at intervention schools up until 6th grade. Participants attended 6th to 10th grade at follow-up. Differences in the homeostasis model assessment of insulin resistance, blood pressure, triglycerides, cholesterol ratios, cardiorespiratory fitness, waist-circumference, and a composite score of these, between participants attending intervention and control schools were analysed by mixed linear regression models. Differences in physical activity at follow-up was analysed cross-sectionally (no baseline available) in *n* = 495.

**Results:**

Compared to controls, children at intervention schools had a non-significant − 0.07 (− 0.32 to 0.18) standard deviations lower composite risk score 6.5 years after project initiation. Likewise, no statistically significant differences between intervention and control schools were found for any of the other outcomes (*p*-values ≥ 0.41). However, six of seven outcomes were in a direction favouring intervention schools. No statistically significant differences between intervention and control schools were observed for physical activity outcomes (*p*-values ≥ 0.13).

**Conclusions:**

An augmented physical activity program including 270 min of weekly physical education provided for three to seven years did not materialize in statistically significant differences in established risk markers in children from intervention compared to control schools. As the intervention was discontinued after 6th grade, the post-intervention effect of augmented physical education throughout adolescence is unknown. School-based physical activity programs may benefit from incorporating instruments for behaviour translation to leisure time in their intervention models to increase the probability of achieving public health relevance.

**Trial registration:**

ClinicalTrials.gov Identifier: NCT03510494.

**Electronic supplementary material:**

The online version of this article (10.1186/s12889-018-5524-4) contains supplementary material, which is available to authorized users.

## Background

Excess adipose tissue accumulation and dysregulated metabolic homeostasis in young people are potentially key markers of increased risk of non-communicable diseases (NCDs) such as cardiovascular disease or type 2 diabetes in adulthood [1–3] and may even predict premature mortality [4]. Physical activity should be considered part of a preventive effort because of its role in energy balance and metabolic regulation [5, 6]. However, trends indicate that physical activity and cardiorespiratory fitness levels in young people may be declining [7–9] and possibly accelerated in low socioeconomic segments of society [9]. These trends are alarming as a lack of physical activity is a leading cause of premature mortality in the adult population [5] and physical activity levels track from childhood to adulthood [10].

Obesity is highly prevalent in nearly all contemporary societies affecting 671 million adults worldwide in 2016 [11] and with a prevalence range of 18 to 38% in European and high-income Western countries [11]. Population-wide initiatives to increase physical activity levels and ameliorate obesity related disorders are therefore warranted [12]. As 70% of obese adults were not obese in their youth [13] preventive actions targeting obese youth will not, even if effective for the individual, prevent the majority of disease burden in the population [14]. School-based approaches have the potential to reach near population-wide coverage as school attendance is mandatory, thus representing an ideal setting for large-scale primordial prevention based on a structural strategy. Structural strategies are appealing as socioeconomic gradients in health inequity may widen with individual-agency approaches [15, 16]. Physical education led by teachers trained in class-management and provision of quality physical education lessons has the potential to provide students with relatively high physical activity levels during classes [17–20], which may carry to higher physical activity patterns on physical education days [18]. Accordingly, provision of additional physical education led by professionals could be a viable option for increasing physical activity levels of school-aged children.

Population-based physical activity interventions initiated in schools (implemented as a physical activity only or part of a multicomponent approach) have shown positive benefits on biological risk factors in young people when evaluated immediately post-intervention [21, 22] However, there is a scarcity of data on long-term benefits on the risk factors [23]. Long-term evaluation is needed to create evidence informed practice. By implementing an augmented school-based physical education program in 2008 the Childhood Health, Activity, and Motor Performance School study DK (CHAMPS study-DK) achieved reductions in clustering of biological risk factors [24] and lowered overweight/obesity prevalence at intervention schools as compared with control schools when evaluated after 2 years [25]. The purpose of this study is to follow-up on these short-term benefits by investigating long-term (6.5 years) differences between children attending control and intervention schools in the CHAMPS-study DK with a focus on; 1) risk factor clustering and 2) single risk-factors.

## Methods

### Setting and study design

**Fig. 1:**
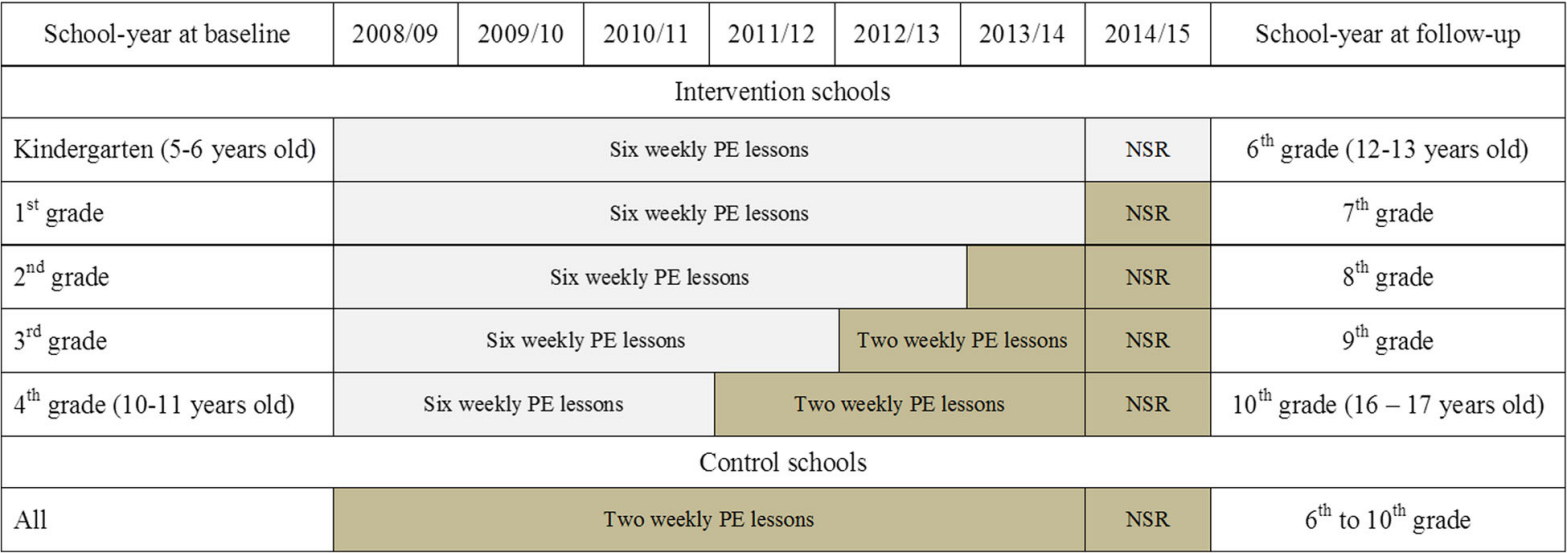
Years of physical education exposure in the CHAMPS-study DK cohort by school-year at baseline and intervention status (2008–2015), Grey boxes indicate six weekly lessons of physical education. Dark boxes indicate two weekly lessons. The NSR mandates 45 min of physical activity on each school-day (not including recess and other breaks). Implementation of the 45 min is managed at the school-level. PE: Physical education, NSR: national school-reform

The CHAMPS-study DK is a natural experiment [26] implemented as a controlled intervention study including children from ten public schools (age range: 5–12 years old) in the municipality of Svendborg, Denmark at inception [17, 27]. The school-years included at baseline are the first year of school (comparable to U.S. kindergarten) to the 4th grade. The CHAMPS-study DK was designed to evaluate a trebling of curricular physical education (from 90 to 270 min per week distributed across at least three school days), initiated by the municipality of Svendborg. All nineteen schools in the municipality were invited to take part in the study of which six schools were willing and able to fund the additional physical educations classes and became intervention schools. Four schools in the municipality, matched on size, rural/urban and sociodemographic uptake area, agreed to serve as controls. Control schools maintained physical education at the national curricular 90 min per week. Based on summary-level statistics from the National Danish Registry of Statistics, parents at schools participating in the project had approximately 15% higher household income, but did not differ in educational level, as compared with non-participating schools [28]. There were no differences in summary-level income or the educational attainment of parents between intervention and control schools [17]. The additional physical education was implemented from the start of the school year in August 2008 and evaluated for effects on biological risk factors and adiposity two years later [24, 25]. After 2010, the six intervention schools have maintained their additional physical education now spanning from kindergarten to the 6th grade (6th grade children approximately 12–14 years old). Thus, from 7th to 9th grade (final mandatory school year in Denmark) the “standard” two physical education lessons per week were provided both at intervention and control schools. Hence, intervention-school participants could receive from three (4th grade in 2008) to seven (Kindergarten in 2008) years of additional physical education (detailed in Fig. 1). A national school-reform mandating 45 min of physical activity on each school day (not including recess and other breaks) was implemented from the beginning of the 2014/2015 school-year. Implementation of the 45 min is managed at the school-level.

### Intervention content

In addition to increased physical education, all physical education teachers (in Denmark physical education is mainly taught by physical education specialists) attended a 40-lesson skill developing course based on an Age-related Training Concept developed by the Danish organization for elite sports (Team Denmark) [29]. The purpose of this program is to augment development of body and motor skills in children and adolescents by considering their physical, physiological, mental and social development. Shortly put, the program is based on play, exercise and games. The focus on technical and coordinative skills was increased as children entered adolescence. Control schools maintained national guidelines. Pertinent to both intervention and control schools is that physical education in Denmark, in addition to physical activity, also has pedagogical and social aims.

### Participants

In 2008, all children and parents from the ten schools (*n* = 1507) were invited to participate in the study [24]. In 2015, all 1278 adolescents attending 6th to 9th grade at the same now nine schools (due to merging of schools) were invited to participate in a follow-up study. Recruitment in 2015 was based on handouts at schools, postal mail of study material to parents, and telephone calls by study staff. Additionally, students who had completed mandatory schooling (“10th^”^ grade in 2015 and 4th grade in 2008) with prior participation in the study (*n* = 179) were sought via postal mail and telephone calls. This study reports long-term effects of the CHAMPS-study DK in 312 participants with 6.5 years of follow-up. Participants included are those providing fasting values of insulin, glucose and lipids in 2008 and 2015 in addition to information on self-reported sexual maturity at both these time-points. Blood chemistry was required for inclusion as these variables constituted the bulk of missing follow-up data and missingness was considered to abundant to impute the variables. Sexual maturity was required because of its key role in insulin-resistance during adolescence [30, 31]. The CHAMPS-study DK was approved by the ethics committee of the region of southern Denmark (S-20080047 and S-20140105) and written informed consent was obtained from a parent or legal guardian at both time-points.

### Outcomes

Data collection for variables used in this study took place during August–October in 2008 (baseline) and February–May in 2015 (follow-up). At both time-points measurements were performed at schools by trained research staff following standardized procedures but not blinded to control/intervention status. Collection of data on 10th grade participants took place in weekends. Blood samples were obtained following an overnight fast between 08.00 and 10.00 am and analysed for total cholesterol (TC), triglyceride, HDL-cholesterol (HDL-c), glucose and insulin in a ISO 9001:2008 certified routine laboratory associated with the University of Vienna, Austria as detailed elsewhere [24]. Briefly, TC, triglycerides, HDL-c and glucose were assessed on a Cobas C System (Roche Diagnostics GmbH, Mannheim, Germany) while insulin was assessed on an Access Immunoassay System (Beckman Coulter, Inc. Brea, CA, USA) using the respective kits. Intermediate precision was determined using human samples and controls in an internal protocol according to the manufacturers. Coefficients of variation for the controls and samples ranged from 0.6 to 0.8% for TC, from 0.6 to 0.9% for triglycerides, from 0.5 to 0.8% for HDL-c, from 0.5 to 0.8% for glucose, 3.1 to 5.6% for insulin. Resting blood pressure was measured using appropriate sized cuffs by a Vital Signs Monitor 300 series with Flexiport™ Blood Pressure *(*Welch Allyn, New York, NY, USA) in 2008 and the Omron 705IT (Omron, Kyoto, Japan) in 2015. Participants sat resting in the sitting position for 5 min before monitoring. At least five subsequent values were recorded with 2-min intervals until the last three values had become stable. The mean of the last three recordings of systolic blood pressure was used in analysis. Waist-circumference was measured by a measurement band (Seca 201, Seca Corporation, Hamburg, Germany) to the nearest 0.5 cm across the umbilical cord following a gentle expiration. At least two measurements were performed with a third undertaken if the two differed by more than 1 cm. Cardiorespiratory fitness was assessed using a field-test (Andersen-test) lasting 10 min with fifteen seconds of intermittent running and pausing. Total distance covered was used to represent cardiorespiratory fitness. Criterion validity (r-squared approximately 0.5 against directly measured maximal oxygen uptake) [32, 33] and test-retest reliability (r-squared approximately 0.7–0.8) of the Andersen-test are acceptable and have been validated in a subsample of the cohort [32].

### Other variables

Body mass was measured to the nearest 0.1 kg on an electronic scale (Tanita BWB-800S, Tanita Corporation, Tokyo, Japan) with participants wearing light clothes. Stature was measured to the nearest 0.5 cm using a portable stadiometer (Seca 214, Seca Corporation, Hamburg, Germany or Harpenden stadiometer (West Sussex, UK)). Both measures were conducted barefoot. Sexual maturity was self-reported by indicating resemblance on five drawings (progressive rating 1–5) of secondary sex characteristics as described by Tanner [34]. Pubic hair was used in boys and breast development in girls. Parents returned mailed questionnaires in 2008 and in 2015. These inquired on the educational attainment of the parents or legal guardians, birthweight of the child, and any (biological) family history of NCDs. When available, data from 2015 was used but in case of non-response or missing answers to the 2015 questionnaire, the 2008 data was used. Physical activity levels were assessed by questionnaires and accelerometry in 2015 only and presented as participation in leisure-time structured physical activity (yes/no), % moderate-to-vigorous physical activity per day (%MVPA/day), and mean counts/minute (protocol and data-reduction details available in Additional file 1.

### Data reduction

The primary outcome of this study is a standardized (mean 0 and standard deviation of 1) composite risk score [35] consisting of the homeostasis model assessment of insulin resistance (HOMA-IR) calculated as (insulin in IU/l x glucose in mmol/l)/22.5) [36], triglyceride, TC:HDLc-ratio, systolic blood pressure, waist-circumference, and cardiorespiratory fitness. A higher composite score represents an unfavourable risk profile. All variables were standardized by age and sex in separate linear regressions and the residuals averaged. Cardiorespiratory fitness was multiplied by − 1 in the composite score. Systolic blood pressure and waist-circumference were additionally standardized for height. Blood chemistry variables in 2015 were additionally standardized for week-day of ascertainment [37] but this information was not available at baseline. Because only 3% of the sample defined themselves in Tanner-category 3–5 at baseline, the sample was re-coded as being either pre-pubertal (stage 1) or pubertal (stages 2–5). At follow-up, 6% defined themselves as Tanner-category 1–2 so this was recoded as stages 1–3, stage 4, or stage 5. Body mass index was calculated as body weight (kg)/stature (meters)^2 and used to define participants according to IOTF weight-status categories [38]. The mother or female guardian’s highest completed education was used as a marker of socioeconomic position [39] and recoded (from an abbreviated seven-level instrument based on a Danish adaptation of the International Standard Classification of Education 2011) to; 0 (no tertiary qualifications) or 1 (any tertiary qualifications). Indication of family history of diabetes (any type), cardiovascular disease (any type) or hypertension in siblings, biological parents or grandparents, were combined into; 0 (no) or 1 (yes). Birthweight was used in continuous form.

### Statistics

Baseline characteristics between intervention and control schools were compared using an unpaired t-test for normal distributed continuous data or Wilcoxon rank-sum test for non-normal distributed data. A chi-squared test was used for categorical data. Details of comparisons between the analytical sample and those lost to follow-up using data obtained in 2008, 2010, 2012, 2013, and 2015 are available in Additional file 2.

#### Statistical analysis of primary and secondary outcomes

Differences between intervention and control schools are presented as composite risk score at follow-up, using control schools as reference, analysed in linear mixed models controlled for baseline values of the respective outcome. In secondary analyses, individual outcomes are analysed using the same approach. Results are presented with 95% confidence intervals (CI). Models additionally included the covariates age, sex, sexual maturity (in 2008 and 2015), educational attainment of the mother or female legal guardian, birthweight, and family history of NCDs. A random intercept for school-class at baseline was included. Random intercepts for school-class or school at follow-up were not included as little variance was explained by these terms. Analysis was performed based on participant’s intervention status at baseline irrespective of their follow-up school membership. Six of ten schools covered kindergarten to 9th grade (no reallocation from primary to secondary school), while four schools (two control and two intervention) included kindergarten to 6th grade only. Of these four schools, students from intervention schools were allocated to other intervention schools, while students from one control school were allocated to an intervention school while students from the other control school were allocated to a school outside of the project. To explore if additional physical education had a distinct effect among those with the least favourable metabolic profiles, participants at control and intervention schools were stratified at the baseline median and analysed separately in secondary analyses. Stratification was performed for each outcome. Physical activity was analysed using the same control variables as the composite risk score but without baseline-adjustment as this data was not available. These analyses used a logistic regression model for structured leisure-time physical activity, and included indicators for the number of total days and weekdays included in models of % MVPA/day and mean counts/min. Participants were included in analysis of physical activity if they; had provided informed consent during the initial phase of the study (2008–2010), at the follow-up in 2015, provided either subjective or objective physical activity data, and had anthropometrical or physical performance data to inform imputation models (*n* = 495, full details available in Additional file 1).

#### Model diagnostics and imputation of missing variables

Linear regression diagnostics were performed by visually inspecting normality and homoscedasticity of model residuals (assumptions met), checking influential observations by calculating the dfbeta (interpretation of results not affected), and visually verifying normal distribution of random intercepts (assumption met). HOMA-IR, triglyceride, TC: HDL-c ratio, waist-circumference and cardiorespiratory fitness were skewed and transformed by the natural logarithm prior to standardization. Missing values (*n* = 3 to 33) of variables other than blood chemistry and sexual maturity were imputed by chained equations (MICE), which is detailed in Additional file 2. As follow-up data is collected as an extension of the original study, no power calculations were performed prior to participant recruitment. TREND and TIDieR checklists are available in Additional file 3: Table S4 and Additional file 4: Table S5. Analysis was conducted in Stata v.15.0 (StataCorp, College Station, TX, USA). A two-sided alpha at the 0.05 level was used to indicate statistical significance.

## Results

### Missing data

**Fig. 2:**
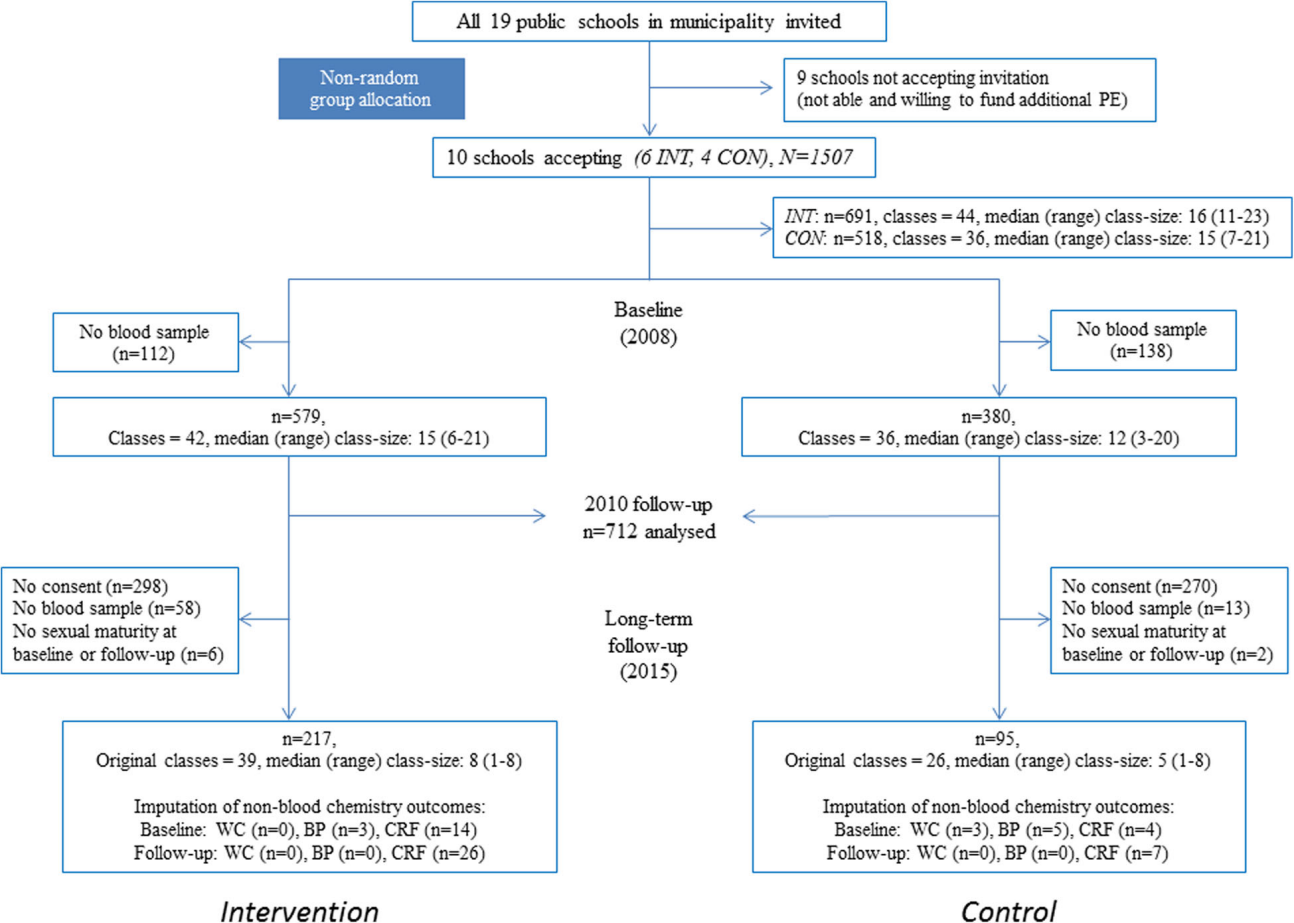
Participant flow-chart, Consenting participants at baseline are lower (1209) than reported in an earlier publication (1218) [24]. INT; intervention, CON; control

In 2008, 1209 students provided informed consent of which a fasting blood sample was available in 959 (64% of invited). In 2015, 745 students (irrespective of previous contact with the study) consented and 580 (40% of invited) provided a fasting blood sample. A total of 312 participants, with a fasting blood sample and information on sexual maturity at both time-points, were available for long-term follow-up (analytical sample: 21% of invited and 33% of those who obtained fasting blood samples in 2008). Of those invited in 2008, 18 and 31% of participants were available for long-term follow-up at control and intervention schools, respectively. Please refer to Fig. 2 for flow-chart as well as in Additional file 2: Table S1 for school-year specific loss to follow-up. The analytical sample differed from their peers unavailable for follow-up, in that the former was younger − 0.75 (− 0.57 to − 0.93) years, and characterized by more favourable anthropometric and cardiorespiratory fitness characteristics (baseline body mass index − 0.40 (− 0.68 to − 0.12) points, waist-circumference − 1.30 (− 2.11 to -0.49) centimetre, and cardiorespiratory fitness 19 (5 to 32) meters. Indications of non-identical missingness characteristics between intervention and control schools were observed for family history of NCDs (*p* = 0.07), educational attainment of the mother or female legal guardian (*p* = 0.03), and pubertal development in 2012 (*p* = 0.02). No evidence to suggest differential missingness characteristics were found for anthropometric variables, the composite risk score, or cardiorespiratory fitness at any time-point (*p*-values > 0.34).

### Characteristics of the analytical sample

Baseline (2008) characteristics of the analytical sample are shown in Table 1. In the analytical sample, 56% of intervention school participants were girls while this was the case for 44% of analysed participants at control schools (p for gender difference = 0.06). At baseline, participants at intervention schools had a 1.79 (0.06 to 3.53) mmHg higher systolic blood pressure, but a 19% lower HOMA-IR score. The prevalence of overweight or obesity was 8.4% in 2008 and 6.8% in 2015 with no difference between intervention and control schools (*p*-values ≥ 0.21).

**Table 1:** Baseline characteristics of participants

	N	Intervention	n	Control	*p*-value for between school-type difference
Age (years)	217	7.8 (1.3)	95	7.8 (1.3)	0.98
Sex (% girls)	217	56	95	44	0.06
Stature (cm)	216	129.2 (8.8)	92	128.8 (9.7)	0.71
Body weight (kg)	217	26.8 (5.4)	92	26.9 (5.9)	0.94
Sexual maturity (% tanner stage 1)	217	75	95	79	0.38
Mothers educational attainment (% any tertiary)	210	65	92	58	0.24
Family history of NCDs (% yes)	211	50	92	53	0.58
Birthweight (gram)	298	3495 (711)	91	3481 (550)	0.87
Composite score (z-scores)	200	-0.07 (0.97)	89	0.17 (1.05)	0.06
Cardiorespiratory fitness (meters)	203	887 (101)	91	885 (107)	0.84
Systolic blood pressure (mmHg)	214	100.1 (6.5)	90	98.3 (8.2)	0.04
Waist-circumference (cm)^a^	217	55.0 (52.5–58)	92	56.5 (53–61.3)	0.08
HOMA-IR^a^	217	0.54 (0.37–0.75)	95	0.65 (0.47–0.89)	0.004
Triglyceride (mmol/l)^a^	217	0.58 (0.46–0.71)	95	0.56 (0.47–0.71)	0.79
TC:HDL-c-ratio^a^	217	2.6 (2.3–3.0)	95	2.6 (2.4–3.0)	0.51

Mean (standard deviation) is given unless otherwise noted

^a^Median (25th–75th centile). *NCD* non-communicable diseases, *HOMA-IR* homeostasis model assessment of insulin resistance, *TC* total cholesterol, *HDL-c* High-density lipoprotein cholesterol

### Primary and secondary outcomes

**Fig. 3:**
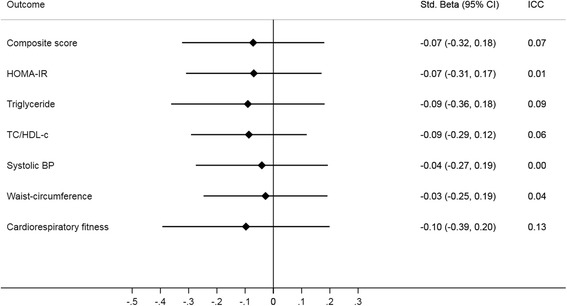
Long-term difference in risk factors between intervention/control schools. Estimates are standardized mean differences at follow-up (in z-scores with 95% CI), using control schools as the reference. Negative values are in favour of intervention schools except for cardiorespiratory fitness where a positive value favours intervention. ICC = intra-class correlation coefficient, BP = blood pressure; TC = total cholesterol, HDL-c = high-density-lipoprotein cholesterol

**Fig. 4:**
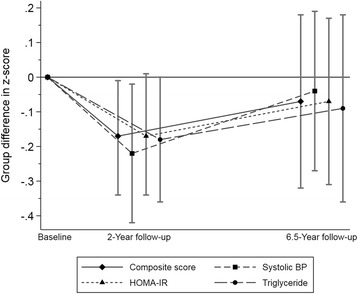
Illustration of difference in biological risk factor development between intervention/control schools in the CHAMPS study-DK (2008, 2010, and 2015). Estimates are mean difference between intervention and control school with a 95% CI. Negative values favour intervention group. Two-year follow-up estimates are from an earlier publication with 712 participants analysed [24]. 6.5 year follow-up are estimates from the 312 participants in this manuscript. Confidence intervals not overlapping zero are statistically significant. Baseline group-differences set to zero as results are presented as adjusted difference in change including baseline-value of outcome, which provides the interpretation that group-mean values are identical at baseline

Figure 3 presents differences in the outcome variables at follow-up between intervention and control schools. The difference in composite risk score did not reach statistical significance (standardized beta with 95% CI) -0.07 (− 0.32 to 0.18). Likewise, differences were non-significant for the individual risk factors (*p*-values ≥ 0.41) with standardized betas ranging from − 0.10 (− 0.39 to 0.20) for cardiorespiratory fitness to − 0.03 (− 0.25 to 0.19) for waist-circumference. All associations, although not significant, favoured intervention schools, except for cardiorespiratory fitness. In un-transformed scales, differences between intervention and control schools were − 0.3 (− 2.1 to 1.5) mmHg, − 0.2 (− 1.6 to 1.2) centimetres, and − 9 (− 39 to 20) meters for systolic blood pressure, waist-circumference and cardiorespiratory fitness, respectively. For blood chemistry variables, differences expressed in untransformed scales were − 0.03 (− 0.12 to 0.06) mmol/l, − 0.08 (− 0.24 to 0.08), and − 0.10 (− 0.33 to 0.14) for triglycerides, TC: HDL-c ratio and HOMA-IR, respectively. Figure 4 depicts a visual illustration of the 2 and 6.5 year (analysed in this study) development of the biological risk factors in the CHAMPS-study DK.

**Fig. 5:**
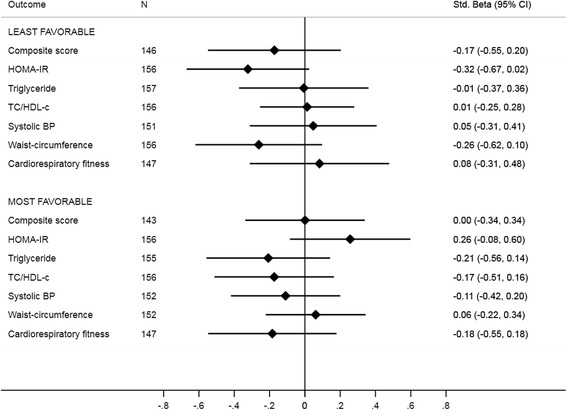
Long-term difference in risk factors between intervention/control, stratified at the median of baseline values. Estimates are standardized mean differences (in z-scores), at follow-up, using control schools as the reference, with 95% CI. Differences are for groups stratified by the respective outcome at baseline (above or below the intervention/control-specific median value). Negative values are in favour of intervention except for cardiorespiratory fitness where a positive value favours intervention. The number of observations differs across outcomes due to missing data at baseline. ICC = intra-class correlation coefficient, BP = blood pressure; TC = total cholesterol, HDL-c = high-density-lipoprotein cholesterol

In analyses with outcomes stratified at the median baseline-level (Fig. 5), the least favourable half at intervention schools presented, compared to the least favourable half at control schools, a − 0.17 (− 0.55 to 0.20) standard deviation lower composite score at follow-up albeit the difference did not reach statistical significance. No statistically significant differences were observed for the individual risk factors. Effect-sizes for (standardized) outcomes stratified at the median ranged from − 0.32 (− 0.67 to 0.02) for HOMA-IR and − 0.26 (− 0.62 to 0.10) for waist-circumference to 0.05 (− 0.31 to 0.41) for systolic blood pressure. When analysing physical activity levels as assessed in 2015 from *n* = 495, neither structured participation in leisure-time physical activity (odds ratio: 0.79 (0.46 to 1.36)), %MVPA/day (unstandardized beta: − 0.17 (− 0.67 to 0.33)), or mean counts/minute (unstandardized beta: − 25 (− 58 to 8)) differed statistically significant between intervention and control schools.

## Discussion

This study evaluated a natural experiment based on a trebling of curricular physical education from 90 to 270 weekly minutes at intervention schools compared to controls. In spite of a demonstrated 2-year efficacy of the intervention, the data did not support sustainability of favourable clustered or single risk factor adaptations comparing intervention with control schools after 6.5 years.

At the 2-year follow-up of the CHAMPS study-DK, the intervention was associated with a favourable composite risk score and lower triglyceride and systolic blood pressure levels [24]. Short-term or immediate post-intervention positive benefits on biological risk factors may be achieved with school-based physical activity interventions [40–44]. It is noticeable that in some studies a positive benefit appears without concomitant effects on adiposity indices [40, 42–44]. Other positive post-intervention benefits of school-based physical activity initiatives may include higher cardiorespiratory fitness [45] and lower obesity levels [46]. Beneficial adaptations are however not universal [47, 48] which calls for identification of those intervention features (or feature combinations) associated with intervention efficacy [49]. Considering the evidence-base as a whole, school-based physical activity interventions suffer from methodological weaknesses particularly in relation to attrition, lack of blinding of outcome assessors, and incomplete description of the randomization process (if relevant) [23]. This study is not exempt from these limitations. The difficulties in achieving strong methodological rigor in the school-based setting are exemplified by the AFLY5-study [50]. This large cluster-randomized controlled trial made substantial efforts in achieving methodological rigor but remained highly susceptible to selection-bias as more than 40% of participants had incomplete data on the main outcome.

### Comparison with existing studies including a longer-term follow-up

Post-interventional successes apart, our data did not support maintained intervention benefits over time. This is in contrast with two similar studies with data on long-term follow-up on biological risk factors, which do provide tentative support of positive sustainable benefits. These studies were based on provision of additional physical education and have evaluated both immediate post-intervention and long-term follow-up difference between intervention and control schools [41, 42, 51]. The CoSCIS study was a non-randomized controlled intervention based on a doubling of weekly physical education from 90 to 180 min for 3 years in addition to provision of outdoor play-ground equipment. The CoSCIS trial observed higher insulin sensitivity in intervention school boys post-intervention and an approximately 3 mmHg lower systolic blood pressure change in boys when followed-up 4 years after the intervention had ceased [42]. No differences between intervention and control school girls were observed at any time point. At the 4-year follow-up of the KISS randomized controlled trial which included nine months of augmented (lesson content) and expanded (from 135 to 225 min weekly) physical education curriculum at its core, participants at intervention schools had a substantially higher cardiorespiratory fitness (5% difference) and greater participation in leisure-time sports compared to participants at control schools [51]. At follow-up, only one risk factor was affected, which was in contrast to the post-intervention evaluation where several metabolic markers (composite score, HDL-cholesterol, triglyceride, glucose), cardiorespiratory fitness, and body composition were favourably modified by the intervention [41]. Long-term differences between intervention and control schools are particularly interesting in the light of the discontinued interventional support in both of these studies. However, even though these sustainable differences would have public health relevance if further maintained over time, statistical significance of results amounts to only one of eleven [42] and one of fourteen [51] investigated outcomes. Thus, in conjunction with high rates of attrition (439 of initially 694 participants [42] and 293 of initially 502 participants [41] available for long-term follow-up, (these losses apart from post-randomization but pre-baseline drop-out)), a cautious interpretation of cause and effect is warranted. Intervention “dose” does not appear to explain the apparent lack of sustained benefits in the CHAMPS-study DK in comparison with the CoSCIS and KISS studies. The CHAMPS-study DK provided an additional 3 h of physical education per week in the intervention group which was double the dose of additional physical educations as delivered in the CoSCIS study. The KISS study was based on daily 45 min of augmented physical education but in comparison with the control condition, only an additional 90 min per week was added. As effects of physical education on physical activity levels do not appear to extend beyond the days when physical education is taking place [18] this could suggest intervention content should be delivered daily. Accordingly, the Sogndal Study was based on 60 daily minutes of physical activity and achieved remarkable effects on biological risk factors [40]. However, a fairly small Icelandic study providing 60 min of daily physical activity in the intervention group did not observe any effect on cardiovascular risk markers after 2 years of intervention [48]. The Cretan Health and Nutrition Education Programme provide further support that sustainable benefits of quality physical activity in school can be achieved. Following 6 years of a comprehensive school health programme (diet, physical activity, and risk behaviours), in which augmented physical education (two lessons per week) was one component, differences between intervention and control schools in blood cholesterol and blood pressure were maintained 4 years post-intervention [52, 53]. Selection bias (attrition and non-randomization) appears the major threat to the validity of the long-term follow-up of the Cretan Health and Nutrition Education Programme. Noticeably, control schools in the Cretan study did not receive any structured physical education for the first half the intervention period which may limit generalizability of the study to a large number of school systems. Conversely, an Australian twenty-week randomized controlled trial including daily twenty minutes fitness sessions did not results in immediate or six-months post-intervention effects on blood pressure or cholesterol levels in high- or low risk children [54]. The internal validity of the Australian study appears robust to selection bias owing to randomization and low attrition. However, the observed lack of short-term effectiveness is potentially explained by the apparently non-trivial time-frame from cessation of the intervention to collection of post-intervention data. Also, the change in BMI appeared larger in intervention group boys as compared to boys in the control condition.

### Public health relevance

Effect sizes presented in this manuscript can be interpreted as standardized effect-sizes (Cohen’s d) which for all outcomes, following Cohen’s suggestions, are interpreted as small (and statistically insignificant). A key question is how large a difference in a population-level risk factor is needed to achieve a meaningful public health impact, and by extension, what is the necessary size of effects from school-based initiatives for this to be considered a viable adjunct to a multisectoral population-wide policy change? In a model-based study, even a 1 mmHg population-wide systolic blood pressure reduction in adults may have a profound effect on the total number of cardiovascular disease cases, surpassing that of reducing the prevalence of uncontrolled hypertension by 10% [55]. As hypertension control programmes operates at sizeable cost [56] and high blood pressure is a leading cause of disability-adjusted life-years [57], even fairly small reductions in blood pressure from school-based approaches are likely public health relevant. Although not a direct contrast between adult hypertension control programmes and augmented school-based physical education, a model-based simulation on U.S. data suggests population-wide augmented physical education in public schools is a cost-effective method for reducing the burden of high blood-pressure attributed cardiovascular disease [58]. When considering the totality of evidence from school-based obesity prevention programs in the general population (including diet, physical activity and combined interventions), two recent meta-analyses reported comparable short term systolic blood pressure reductions of 1.64 (95% CI: 2.56 to 0.71) [22] and 1.95 (2.85 to 1.05) [59] mmHg. Our long-term follow-up did not support such effect-sizes as the systolic blood pressure at intervention schools was 0.22 mmHg lower (statistically insignificant) as compared to control schools.

### Do physical activity interventions increase overall physical activity levels in young people?

The CHAMPS-study DK intervention was associated with relatively high physical activity levels during physical education [17]. No differences in mean physical activity intensity or percentage of time spent at MVPA during physical education were observed between intervention and control schools but the volume delivered was, by design, higher at intervention schools. This suggests the aim of increasing the volume of physical activity in school was accomplished and the intervention thereby implemented satisfactory [17]. Part of the intervention was based on teacher training focusing on an age-related physical education content. Unfortunately we do not have data on qualitative features of delivered content besides time spent above established physical activity intensity thresholds. We note that in Denmark physical education teachers are content-professionals and not e.g. class-room teachers. Thereby quality physical education is also expected at control schools. Despite the appearance of intervention effects on school-time physical activity levels and achievement of favourable metabolic profiles at intervention schools, no differences in total physical activity levels between intervention and control schools were evident during the first 2 years of the study [17]. Similarly, in the cross-sectional analysis of physical activity levels in 2015, which unfortunately lacked baseline data to provide a longitudinal interpretation, we did not observe different physical activity levels at intervention schools as compared to control. As the additional physical education lessons were discontinued after the 6th grade it is possible that the lack of long-term differences between intervention and control schools may owe to the intervention not having resulted in the necessary motivation and/or skills to compensate for the removal of physical education lessons (e.g. by intervention schools students increasing out-of-school physical activity). The creation of lasting habits is likely necessary for school-based initiatives to result in meaningful health benefits. In this light, strictly school-based interventions build solely on increased provision of structured physical activity but without an instrument for translation of behaviour into discretionary time may, although efficacious in increasing physical activity when the intervention is delivered [17, 18, 60], not provide the necessary impetus to increase physical activity external to the intervention. Reductions in leisure-time sports participation at intervention schools is a potential co-intervention from adding additional school hours and physical education classes. As sports participation is associated with a favourable risk factors profile [61] the direction of such a hypothetical reduction in physical activity in discretionary time would likely be towards a zero effect of the intervention. Our analysis of group means did not support the occurrence of such an effect, but this does not preclude unintended co-interventions in some sub-groups of the sample. In the short term, particularly promising effects of school-based interventions including a family component have previously been highlighted. This could relate to a supportive environment [45, 62, 63] for adapting leisure-time physical activities and thus formation of lasting habits. Data to facilitate short-term intervention efficacy is increasing [49] but without evaluation of which intervention components associates with long-term behavior modification, information to guide public health policy will remain limited. Taken together, there have been limited identification of factors (or their combinations) leading to efficacious interventions [49, 64] and particularly to sustainability of achieved success over time [64]. With regards to physical activity levels evidence from twelve randomized controlled trials in youth above 10 years of age in fact suggests objectively measured overall physical activity levels are not increased by current school-based interventional approaches, although some heterogeneity exists [65]. A review also including subjective physical activity assessments came to the reverse conclusion, but given the possibility of recall and social-desirability bias in self-reported data and the generally unconvincing quality of included studies, these results should be interpreted with caution [23]. In a comprehensive meta-analysis pooling thirty studies including an objective physical activity assessment (accelerometry only) increases in physical activity from interventions were in the order of 4 min of MVPA/day [66]. The results of two recent meta-analyses of interventions including a “long-term” follow-up, defined as minimum 6 months and 4 weeks post-intervention, both conclude there is a lack of evidence to suggest sustainable effects on physical activity levels [67, 68]. The apparent lack of even short-term effects on physical activity is in contrast to interventional effects on the biological risk factors and may relate to measurement error and larger attrition rates for physical activity outcomes. Alternatively, changes in dietary behaviours as a consequence of the intervention (intended or not), may explain the discrepancy.

### Limitations

The present study should be interpreted in the light of several limitations. The non-randomized design precludes a causal interpretation, and although matching of schools was performed, the possibility of selection bias remains. Analyses were adjusted for potential demographic and biological confounders but information on e.g. nutritional behaviours was not available. Thereby potential confounding from dietary habits or other unmeasured and unbalanced variables cannot be eliminated. Selection bias may also be introduced as missing data was frequent, particularly for the oldest participants. Comprehensive analyses comparing included and non-included individuals at baseline and over time revealed favourable anthropometric and cardiorespiratory fitness profiles in the included sample as compared to the sample lost to follow-up. This could have reduced the potential for beneficial intervention-related adaptations to occur as, in the 2-year evaluation of the study, particularly strong effects were observed in the half presenting the least favourable metabolic profile [24]. Importantly, no evidence of differential missingness characteristics in these variables across intervention and control participants was observed albeit this does not eliminate the possibility of selection bias. Because the intervention was embedded in the school curriculum it was not possible to collect true pre-intervention data. Instead this was obtained up to 2 months after intervention initiation. Accordingly, differences in baseline clinical characteristics, and particularly insulin sensitivity, between intervention and control participants could be the result of early adaptations to the increased physical education lessons. If this is the case, controlling for baseline values could be considered conservative [69]. When analyses of follow-up HOMA-IR and composite-score were repeated without including the baseline values, coefficients were roughly doubled (in favour of intervention), but remained statistically insignificant (data not shown). Although the available sample size was similar to previous studies with a long-term follow-up, it must be considered low with only 95 participants in the control group available. Hence, the possibility of a type II error cannot be ruled out. Sample size consideration was also the reason no subgroup analysis according to years of intervention exposure was pursued. Multiple imputation was used to retain sample-size, but the number of individuals with missing data on blood samples at follow-up was too large to meaningfully impute this information. The implementation of a nationwide school-reform mandating 45 min of physical activity on each school day from 2014 could reduce heterogeneity in the participating school children’s exposure to physical activity. This could potentially mask effects of an earlier intervention by affecting activity patterns at both control and intervention schools. However, when performing research within the educational system, and particularly when including a prolonged follow-up period, changes to school-policies or the school environment (e.g. ban of purchasing sugar-sweetened beverages at schools, new safer roads for active commuting, new playground equipment) mandated at the school or national level is likely the norm. Such structural changes exemplifies the difficulties in conducting research in an uncontrolled environment were strict adherence to study-conditions are not possible. Finally, because the municipality of Svendborg considered the intervention to be highly successful, in 2012 the additional physical education was implemented at all schools in the municipality (including control schools). However, because the additional physical education was provided to children starting in kindergarten from the school year 2012, children serving as controls in this study would not be directly affected.

## Conclusions

Despite 2-year intervention efficacy, a trebling of curricular physical activity from kindergarten to 6th grade did not result in statistically significant reductions in clustered or single biological risk factors between intervention and control schools, when evaluated after 6.5 years of follow-up. Future research featuring school-based physical activity interventions should 1) pre-plan for long-term follow-up, 2) actively seek to minimize attrition over time, and 3) incorporate instruments for behaviour translation to leisure time into their physical activity intervention models to increase the probability of long-term effects on population health.

## Additional files

Additional file 1: Description of methodology used for physical activity assessment, data-reduction, and imputation of missing variables in cross-sectional analysis of follow-up physical activity levels. (PDF 345 kb)

Additional file 2: Information on multiple imputation procedure, missing data by school-year (Table S1), and comparison between participants available for long-term follow-up and those unavailable for follow-up (**Table S2** and **S3**). (PDF 382 kb)

Additional file 3: TREND checklist in **Table S4**. (PDF 435 kb)

Additional file 4: TIDier checklist in **Table S5**. (PDF 464 kb)

## Abbreviations

CHAMPS-study DK: Childhood Health, Activity, and Motor Performance School Study Denmark
CI: Confidence interval
HDL-c: High-density-lipoprotein cholesterol
HOMA-IR: Homeostasis model assessment of insulin resistance
MVPA: Moderate-to-vigorous physical activity
NCD: Non-communicable disease
TC: Total cholesterol
