# Supplementary material for: Long-term follow-up on biological risk factors, adiposity, and cardiorespiratory fitness development in a physical education intervention: a natural experiment (CHAMPS-study DK)
Source: BMC Public Health. 2018 May 9;18:605. doi: 10.1186/s12889-018-5524-4 (PMC5941623; doi:10.1186/s12889-018-5524-4)
Supplement: Supplementary file 1 — Description of methodology used for physical activity assessment, data-reduction, and imputation of missing variables in cross-sectional analysis of follow-up physical activity levels. (PDF 345 kb) [file 12889_2018_5524_MOESM1_ESM.pdf]

Additional File 1. Physical activity assessment

Physical activity was assessed by a questionnaire and accelerometry in 2015. Participants were included in analysis of physical activity if they; had provided informed consent during the initial phase of the study (2008-2010), at the follow-up in 2015, provided either subjective or objective physical activity data, and had anthropometrical or physical performance data to inform imputation models. This left 495 participants for analysis. Physical activity data was only analysed cross-sectionally as no appropriate baseline data was available (see [1] for details of physical activity assessment in the CHAMPS-study DK). Questionnaires were completed by participants at schools under the supervision of research staff. Students were asked to indicate their participation in structured leisure-time physical activity. Answers were dichotomized as yes/no. Hip-mounted accelerometers (Actigraph GT3X and GT3X+, Pensacola, FL, USA) were distributed allowing for a minimum of seven consecutive days of measurement. Accelerations were recorded in 30 Hz (GT3X+) or 2 seconds (GT3X – due to memory limitations) epoch, but data was downloaded using a 10-second epoch. Accelerometers were set to start recording at 06:00 on the day after participants received the device, with participants instructed to remove the device only when performing aquatic activities, showering and at night. Because missing data was expected, we issued a screening protocol to maximize data availability. Participants not providing at least four days with ten hours of wear-time including at least one weekend day were asked to re-wear the monitor (no differences in wear-time, moderate physical activity or vigorous physical activity (p-values >0.10) were observed between participants providing sufficient data at the first distribution of accelerometers and those providing sufficient data at the re-wear period). The measurement period lasted from February to May 2015. The percentage of participants meeting the screening criterion increased from 57 to 75 % of eligible participants after including re-wear. Accelerometer data was analyzed using open-source software (Propero, University of Southern Denmark, Odense, Denmark).

Consecutive strings of zero counts of ≥60 min were considered "monitor not worn" and discarded from summation of physical activity and wear-time. Accept criteria for analysis were set at three days (not requiring a weekend-day) of at least 8 h of worn time collected from 06:00 to 24:00. Data from the first distribution period and the potential re-wear period was included if available. Days with vigorous physical activity > 3 standard deviations of the mean were discarded to remove influence from potential accelerometry malfunctions (e.g. vigorous physical activity for >15 % of the day). Physical activity was expressed as mean counts/min and %MVPA/day, with MVPA defined as counts/min ≥2296 counts/min [2] but rescaled to match the 10 seconds epoch. Physical activity levels in 2015, comparing intervention with control schools, were analysed using linear and logistic mixed regression models for %MVPA/day and sport-participation, respectively. Models were controlled for age, sex, sexual maturity (Tanner stages), educational attainment of the mother or female guardian, family history of NCD's, and including a random intercept for school-class membership in 2015. Accelerometry data was further controlled for number of included days, and number of included weekend-days.

Multiple imputation by chained equations (MICE) was used to impute missing data (n=29 for %MVPA/day and n=5 for leisure time sports or physical activity) using 2015 information on stature, body weight, waist-circumference, cardiorespiratory fitness, systolic blood pressure, sexual maturity, blood-sample available (yes/no), number of valid total and weekend accelerometry days, school membership and accelerometry/questionnaire data. Birthweight, educational attainment of the mother or female guardian, and family history of NCD's from the 2015 parental questionnaire was used and updated with the 2008 questionnaire if missing. Coefficients end their standard errors were based on twenty imputed datasets. Imputation models were visually checked for convergence and the reproducibility of the estimates where inspected by Monte Carlo errors. The imputations are

based on the assumption of data being missing at random conditional on the observed variables (MAR).

In non-imputed data (n=466), the median (25-75th percentile) included days and weekend-days were 7 (6 – 8) and 2 (1 – 2) at intervention and 7 (6 – 7) and 2 (1 – 2) at control schools, respectively with no statistically significant difference between schools types (p-values ≥0.36). Median (25-75th percentile) wear-time was 13.7 (13.0 – 14.3) and 13.7 (13.0 – 14.4) hours at intervention and control schools, respectively with no statistically significant difference between school types (p = 0.97).

References

1. Moller N, Tarp J, Kamelarczyk E, Brond J, Klakk H, Wedderkopp N: Do extra compulsory physical education lessons mean more physically active children - findings from the childhood health, activity, and motor performance school study Denmark (The CHAMPS-study DK). *Int J Behav Nutr Phys Act* 2014, 11:121.
2. Trost SG, Loprinzi PD, Moore R, Pfeiffer KA: Comparison of accelerometer cut points for predicting activity intensity in youth. *Med Sci Sports Exerc* 2011, 43:1360-1368.
