# Supplementary material for: Long-term follow-up on biological risk factors, adiposity, and cardiorespiratory fitness development in a physical education intervention: a natural experiment (CHAMPS-study DK)
Source: BMC Public Health. 2018 May 9;18:605. doi: 10.1186/s12889-018-5524-4 (PMC5941623; doi:10.1186/s12889-018-5524-4)
Supplement: Supplementary file 2 — Information on multiple imputation procedure, missing data by school-year (Table S1), and comparison between participants available for long-term follow-up and those unavailable for follow-up (Table S2 and S3). (PDF 382 kb) [file 12889_2018_5524_MOESM2_ESM.pdf]

Additional file 2. Missing data

TABLE OF CONTENTS

Page 1. Detailed approach for handling missing variables other than blood chemistry and sexual maturity. Multiple imputation using chained equations (MICE)

Page 2. Table S1. School-year specific and total loss-to-follow-up stratified by intervention and control schools

Page 2. Details of methods used for drop-out comparison. Comparison of participants available for long-term follow-up with participants unavailable for analysis

Page 4. Table S2. Adjusted differences between analysed and non-analysed participants by year of data availability.

Page 7. Table S3. Descriptive characteristics of analysed and non-analysed samples by year of data availability.

Multiple imputation using chained equations

Missing values of variables other than blood chemistry and sexual maturity were imputed by chained equations (MICE). Beta coefficients and standard errors were obtained based on twenty imputed datasets. Imputation models were visually checked for convergence and the reproducibility of the estimates where inspected by Monte Carlo errors. The imputations are based on the assumption of data being missing at random conditional on the observed variables (MAR). Imputations were based on all variables included in analytical models except an indicator for school was used instead of class and the addition of stature, body weight and waist-circumference in 2010 as data was near complete for these variables. The number of imputed observations ranged from three (waist-circumference in 2008) to 33 (cardiorespiratory fitness in 2015). Data on sexual maturity could not be imputed due to non-convergence of models, hence participants not having this information were considered lost to follow-up (n=8).

Table S1. School-year specific and total loss-to-follow-up stratified by intervention and control schools

	School-year at follow-up					
	Year 6	Year 7	Year 8	Year 9	Year 10	Total
**Intervention schools (n)**	103	124	122	120	110	579
Analysed (% of n)	54	47	45	32	9	37
Non-analysed (% of n)	46	53	55	68	91	63
**Control schools (n)**	47	76	79	87	91	380
Analysed (% of n)	60	28	32	17	7	25
Non-analysed (% of n)	40	72	68	83	93	75

Numbers (n) refers to the 959 participants with a fasting blood sample at baseline. Percentages are calculated within intervention/control columns.

Comparison of participants available for long-term follow-up with participants unavailable for analysis in the CHAMPS-Study DK

Table S2 and S3 presents comparisons of the sample included in the manuscript (analysed sample) with participants from the CHAMPS-study DK not available for long-term evaluation of biological risk factors. The non-analysed group includes participants with (n= 647) and without (n= 250) data on fasting blood samples at baseline. Comparisons are made using data from 2008, 2010, 2012, 2013, and 2015 CHAMPS- Study DK data collections [1, 2]. Comparison of characteristics between the analysed sample and those with missing data was conducted by mixed effects linear or logistic regression, as applicable, including participant age and a random intercept for school-class. In secondary analyses, evidence of non-identical characteristics between non-analysed participants at intervention and control schools is investigated by adding an evaluating an intervention arm (coded as 0 or 1)-by-availability of follow-up data (coded as 0 or 1) interaction term. Estimates are presented as

unstandardized betas (1=analysed sample) for continuous variables or prevalence for categorical variables unless indicated otherwise. Models are adjusted for age and a random intercept for school-class. Family history of cardiovascular disease, hypertension, or diabetes, birthweight, mother or female guardian's educational attainment, and body mass index of the mother are included from the baseline questionnaire only.

References to data sources

1. Klakk H, Andersen LB, Heidemann M, Moller NC, Wedderkopp N: Six physical education lessons a week can reduce cardiovascular risk in school children aged 6-13 years: a longitudinal study**.** *Scand J Public Health* 2014, 42**:**128-136.
2. Junge T, Larsen LR, Juul-Kristensen B, Wedderkopp N: The extent and risk of knee injuries in children aged 9-14 with Generalised Joint Hypermobility and knee joint hypermobility - the CHAMPS-study Denmark**.** *BMC Musculoskelet Disord* 2015, 16**:**143.

Table S2. Adjusted differences between analysed and non-analysed participants in the CHAMPS-Study DK, by year of data availability.

	N (non-analysed)	N (analysed)	Mean difference (95% CI) OR crude prevalence (1=analysed OR first number= not in sample)	p-value[a]
Gender (% girls)	897	312	53 vs 52	0.99
Age (years)	897	312	-0.75 (-0.57 to -0.93)	<0.001
**From 2008 questionnaire**				
Mothers BMI (kg/m^2)	712	279	-0.25 (-0.82 to 0.33)	0.40
Family history of CVD, diabetes, hypertension (yes - %)	747	289	38 vs 38	0.99
Birthweight (grams)	605	239	-81 (-183 to 20)	0.12
Educational attainment of mother (% any tertiary)	729	285	43 vs. 48	0.15
**2008**				
Stature (cm)	865	308	-0.54 (-1.30 to 0.20)	0.16
Body weight (kg)	863	309	-0.99 (-1.69 to -0.29)	0.005
BMI (kg/m^2)	863	308	-0.40 (-0.68 to -0.12)	0.004
Sexual maturity[b]	835	312	0.94 (0.62 to 1.41)	0.76
Waist-circumference (cm)	862	309	-1.30 (-2.11 to -0.49)	0.002
Cardiorespiratory fitness (meters)	827	294	18 (5 to 32)	0.007
Systolic blood pressure (mmHg)	835	304	-0.79 (-1.74 to 0.16)	0.10
Composite score[c]	647	312	-0.05 (-0.19 to 0.09)	0.47
**2010**				
Stature (cm)	800	309	-0.52 (-1.38 to 0.34)	0.24
Body weight (kg)	799	309	-1.10 (-2.03 to -0.17)	0.02
BMI (kg/m^2)	799	309	-0.38 (-0.69 to 0.06)	0.02
Sexual maturity[b]	781	305	0.91 (0.66 to 1.27)	0.59

Waist-circumference (cm)	800	309	-0.96 (-1.94 to 0.01)	0.053
Cardiorespiratory fitness (meters)[d]	698	271	27 (13 to 41)	<0.001
Systolic blood pressure (mmHg)	800	309	-0.14 (-1.16 to 0.88)	0.79
Composite score[c]	607	269	-0.07 (-0.22 to 0.08)	0.36
**2012**				
Stature (cm)	579	287	-0.45 (-1.42 to 0.53)	0.37
Body weight (kg)	575	286	-1.65 (-2.77 to -0.53)	0.004
BMI (kg/m^2)	575	286	-0.55 (-0.90 to 0.20)	0.002
Sexual maturity[e]	574	284	0.76 (0.57 to 1.02)	0.07
Waist-circumference (cm)	576	286	1.59 (-2.68 to -0.50)	0.004
Cardiorespiratory fitness (meters)	484	246	22 (6 to 38)	0.008
**2013**				
Stature (cm)	479	272	0.06 (-1.05 to 1.17)	0.92
Body weight (kg)	475	272	-1.16 (-2.44 to 0.12)	0.08
BMI (kg/m^2)	475	272	-0.43 (-0.80 to -0.06)	0.02
Sexual maturity[e]	477	272	0.95 (0.68 to 1.33)	0.76
Waist-circumference (cm)	472	267	-1.17 (-2.27 to -0.07)	0.04
Cardiorespiratory fitness (meters)	396	236	18 (1 to 36)	0.04
**2015**				
Stature (cm)	312	150	0.42 (-1.14 to 1.99)	0.60
Body weight (kg)	311	150	-1.12 (-3.04 to 0.78)	0.25
BMI (kg/m^2)	311	150	-0.44 (-0.96 to 0.08)	0.10
Sexual maturity[e]	312	149	1.02 (0.64 to 1.61)	0.94
Waist-circumference (cm)	312	150	-1.18 (-2.71 to 0.34)	0.13
Cardiorespiratory fitness (meters)	279	119	20 (-4 to 44)	0.10
Systolic blood pressure (mmHg)	308	148	-0.22 (-1.85 to 1.42)	0.79

Composite score[c]	312	97	-0.29 (-0.52 to -0.06)	0.01

OR: odds ratio, BMI: body mass index; CVD; cardiovascular disease,

Estimates are unstandardized (unless noted otherwise) betas (0=not in sample) adjusted for age and a random intercept for school-class OR absolute prevalence (first number= not in sample)

[a]P is for between-sample difference

[b]Mixed effects logistic regression – odds ratio of being >stage 1

[c]Composite scores is standardized and includes standardized values of lnHOMA-IR, lnTriglyceride and lnTC:HDLc ratio

[d]using data from spring 2011 (6 months later) as cardiorespiratory fitness was not assessed fall 2010

[e]Mixed effects ordinal logistic regression – odds ratio of being in a higher tanner stage

Table S3. Investigation of non-identical missingness characteristics at intervention and control schools by year of data availability.

	Control		Intervention		p-value[a]
	non-analysed	analysed	non-analysed	analysed	
Sex (% girls)[b]	48	56	46	44	0.18
Age in 2008 (years)	8.6 (1.5)	7.8 (1.3)	8.6 (1.4)	7.8 (1.3)	0.91
**From 2008 questionnaire**					
Mothers BMI (kg/m^2)	24.0 (3.9)	24.0 (3.7)	24.4 (4.1)	24.0 (4.0)	0.66
Family history of CVD, Diabetes, hypertension (% yes)[b]	38	47	39	35	0.07
Birthweight (grams)	3582 (623)	3481 (557)	3506 (675)	3468 (688)	0.55
Educational attainment of mother (% any tertiary)[b]	48	43	38	50	0.03
**2008**					
Stature (cm)	133.6 (10.1)	128.8 (9.7)	134.0 (9.7)	129.2 (8.8)	0.95
Body weight (kg)	30.1 (7.3)	26.9 (5.9)	30.2 (7.0)	26.8 (5.4)	0.92
BMI (kg/m^2)	16.7 (2.2)	16.0 (1.7)	16.6 (2.3)	15.9 (1.7)	0.71
Sexual maturity (% > tanner stage 1)[c]	31	25	36	21	0.09
Waist-circumference (cm)	59.4 (7.0)	57.2 (5.5)	59.1 (7.0)	56.1 (5.6)	0.78
Cardiorespiratory fitness (meters)	890 (112)	885 (107)	895 (107)	887 (101)	0.79
Systolic blood pressure (mmHg)	101.5 (8.1)	98.3 (8.2)	102.0 (7.9)	100.1 (6.5)	0.79
Composite score[d]	0.03 (0.99)	0.14 (0.94)	-0.01 (1.02)	-0.12 (0.97)	0.37
**2010**					
Stature (cm)	145.4 (10.5)	140.6 (10.2)	146.4 (10.5)	141.2 (9.1)	0.65
Body weight (kg)	37.6 (9.0)	33.9 (8.0)	38.1 (9.2)	33.8 (6.9)	0.57
BMI (kg/m^2)	17.6 (2.4)	16.9 (2.2)	17.5 (2.6)	16.8 (2.0)	0.89
Sexual maturity (% > tanner stage 1)[c]	74	67	76	62	0.25
Waist-circumference (cm)	64.1 (8.4)	61.5 (7.4)	64.4 (8.2)	61.3 (6.9)	0.62

Cardiorespiratory fitness (meters)[e]	978 (103)	976 (84)	980 (113)	977 (99)	0.79
Systolic blood pressure (mmHg)	102.8 (7.8)	102.3 (7.6)	101.6 (8.2)	100.3 (7.4)	0.34
Composite score[d]	0.07 (1.03)	0.13 (1.13)	-0.03 (0.96)	-0.10 (0.95)	0.52
**2012**					
Stature (cm)	152.3 (10.6)	149.4 (11.3)	154.6 (10.8)	149.8 (9.7)	0.72
Body weight (kg)	43.1 (10.5)	39.4 (8.9)	44.4 (10.5)	39.7 (8.4)	0.94
BMI (kg/m^2)	18.4 (2.7)	17.5 (2.3)	18.3 (2.7)	17.5 (2.1)	0.62
Sexual maturity (% stage 1, 2, 3, 4+5)[f]	19 / 36 / 31 / 14	21 / 41 / 29 / 9	16 / 29 / 33 / 22	28 / 40 / 25 / 7	0.01
Waist-circumference (cm)	68.5 (8.6)	65.7 (6.8)	67.2 (8.1)	64.2 (7.4)	0.68
Cardiorespiratory fitness (meters)	1010 (118)	1022 (127)	1022 (106)	1021 (97)	0.97
**2013**					
Stature (cm)	157.2 (11.0)	155.3 (12.1)	160.8 (10.7)	156.3 (10.1)	0.48
Body weight (kg)	46.3 (10.7)	43.9 (10.9)	50.0 (11.5)	44.7 (9.1)	0.92
BMI (kg/m^2)	18.5 (2.5)	18.0 (2.5)	19.1 (2.9)	18.1 (2.1)	0.81
Sexual maturity (% stage 1+2, 3, 4+5)[f]	40 / 33 / 36	45 / 28 / 27	29 / 28 / 43	46 / 35 / 19	0.22
Waist-circumference (cm)	68.6 (7.8)	66.8 (7.3)	70.5 (8.4)	67.4 (6.7)	0.94
Cardiorespiratory fitness (meters)	1010 (109)	1023 (110)	1034 (123)	1026 (114)	0.98
**2015**					
Stature (cm)	163.9 (10.1)	166.4 (10.8)	166.7 (9.3)	167.4 (9.3)	0.74
Body weight (kg)	53.0 (12.0)	54.6 (13.2)	56.2 (11.6)	54.9 (9.8)	0.86
BMI (kg/m^2)	19.5 (2.8)	19.5 (3.0)	20.1 (3.2)	19.5 (2.4)	0.68
Sexual maturity (% stage 1-3, 4, 5)[f]	52 / 44 / 4	41 / 43 / 16	40 / 44 /15	39 / 48 / 13	0.81
Waist-circumference (cm)	72.3 (9.0)	72.2 (8.3)	73.0 (9.3)	71.3 (7.1)	0.70
Cardiorespiratory fitness (meters)	1078 (110)	1119 (112)	1075 (113)	1096 (112)	0.75
Systolic blood pressure (mmHg)	106.9 (8.4)	107.4 (8.8)	107.5 (9.7)	107.6 (8.4)	0.63
Composite score[d]	0.25 (0.87)	0.08 (1.17)	0.21 (1.21)	-0.14 (0.86)	0.63

OR: odds ratio, BMI: body mass index; CVD; cardiovascular disease,

Estimates are unstandardized betas adjusted for age and a random intercept for school-class unless indicated otherwise.

[a]p-value is from intervention arm-by-availability of follow-up data interaction term

[b]Logistic regression model

[c]Mixed effects logistic regression model

[d]Composite scores is standardized and includes standardized values of lnHOMA-IR, lnTriglyceride and lnTC:HDLc ratio

[e]using data from spring 2011 (6 months later) as cardiorespiratory fitness was not assessed fall 2010

[f]Mixed effects ordinal logistic regression model
