# Supplementary material for: Long-term follow-up on biological risk factors, adiposity, and cardiorespiratory fitness development in a physical education intervention: a natural experiment (CHAMPS-study DK)
Source: BMC Public Health. 2018 May 9;18:605. doi: 10.1186/s12889-018-5524-4 (PMC5941623; doi:10.1186/s12889-018-5524-4)
Supplement: Supplementary file 3 — TREND checklist in Table S4. (PDF 435 kb) [file 12889_2018_5524_MOESM3_ESM.pdf]

Table S4. TREND Statement Checklist

Paper Section/Topic	Item No.	Descriptor	Reported? ✓	Pg #
**TITLE and ABSTRACT**				
Title and Abstract	1	• Information on how units were allocated to interventions		Abstract
		• Structured abstract recommended		Abstract
		• Information on target population or study sample		Abstract
**INTRODUCTION**				
Background	2	• Scientific background and explanation of rationale		Introduction
		• Theories used in designing behavioral interventions		None applied
**METHODS**				
Participants	3	• Eligibility criteria for participants, including criteria at different levels in recruitment/sampling plan (e.g., cities, clinics, subjects)		Setting and study design + Statistics
		• Method of recruitment (e.g., referral, self-selection), including the sampling method if a systematic sampling plan was implemented		Setting and study design
		• Recruitment setting		Setting and study design
		• Settings and locations where the data were collected		Outcomes
Interventions	4	• Details of the interventions intended for each study condition and how and when they were actually administered, specifically including:		
		○ Content: what was given?		Setting and study design + Table 1
		○ Delivery method: how was the content given?		Setting and study design + Intervention content
		○ Unit of delivery: how were subjects grouped during delivery?		Setting and study design
		○ Deliverer: who delivered the intervention?		Setting and study design + Intervention content
		○ Setting: where was the intervention delivered?		Setting and study design + Intervention content
		○ Exposure quantity and duration: how many sessions or episodes or events were intended to be delivered? How long were they intended to last?		Setting and study design + Table 1
		○ Time span: how long was it intended to take to deliver the intervention to each unit?		Setting and study design + Table 1
		○ Activities to increase compliance or adherence (e.g., incentives)		
Objectives	5	• Specific objectives and hypotheses		Introduction
Outcomes	6	• Clearly defined primary and secondary outcome measures		Data reduction
		• Methods used to collect data and any methods used to enhance the quality of measurements		Methods
		• Information on validated instruments such as psychometric and biometric properties		Methods

Table S4. TREND Statement Checklist

Sample size	7	• How sample size was determined and, when applicable, explanation of any interim analyses and stopping rules		Setting and study design + Statistics
Assignment method	8	• Unit of assignment (the unit being assigned to study condition, e.g., individual, group, community)		Setting and study design
		• Method used to assign units to study conditions, including details of any restriction (e.g., blocking, stratification, minimization)		Setting and study design
		• Inclusion of aspects employed to help minimize potential bias induced due to non-randomization (e.g., matching)		Setting and study design
Blinding (masking)	9	• Whether or not participants, those administering the interventions, and those assessing the outcomes were blinded to study condition assignment; if so, statement regarding how the blinding was accomplished and how it was assessed		Setting and study design + Outcomes
Unit of Analysis	10	• Description of the smallest unit that is being analyzed to assess intervention effects (e.g., individual, group, or community)		Statistics
		• If the unit of analysis differs from the unit of assignment, the analytical method used to account for this (e.g., adjusting the standard error estimates by the design effect or using multilevel analysis)		Statistics
Statistical methods	11	• Statistical methods used to compare study groups for primary methods outcome(s), including complex methods for correlated data		Statistics
		• Statistical methods used for additional analyses, such as subgroup analyses and adjusted analysis		Statistics
		• Methods for imputing missing data, if used		Statistics
		• Statistical software or programs used		Statistics
**RESULTS**				
Participant flow	12	• Flow of participants through each stage of the study: enrollment, assignment, allocation and intervention exposure, follow-up, analysis (a diagram is strongly recommended)		Figure 1
		○ Enrollment: the numbers of participants screened for eligibility, found to be eligible or not eligible, declined to be enrolled, and enrolled in the study		Figure 1 + Setting and study design + Participants
		○ Assignment: the numbers of participants assigned to a study condition		Figure 1
		○ Allocation and intervention exposure: the number of participants assigned to each study condition and the number of participants who received each intervention		Figure 1
		○ Follow-up: the number of participants who completed the follow-up or did not complete the follow-up (i.e., lost to follow-up), by study condition		Figure 1 + Participants + Statistics
		○ Analysis: the number of participants included in or excluded from the main analysis, by study condition		Statistics + Figure 1
		• Description of protocol deviations from study as planned, along with reasons		
Recruitment	13	• Dates defining the periods of recruitment and follow-up		Outcomes
Baseline data	14	• Baseline demographic and clinical characteristics of participants in each study condition		Table 2
		• Baseline characteristics for each study condition relevant to specific disease prevention research		Table 2
		• Baseline comparisons of those lost to follow-up and those retained, overall and by study condition		Additional file 2
		• Comparison between study population at baseline and target population of interest		Not applied
Baseline equivalence	15	• Data on study group equivalence at baseline and statistical methods used to control for baseline differences		Sample characteristics + Statistics
Numbers analyzed	16	• Number of participants (denominator) included in each analysis for each study condition, particularly when the denominators change for different outcomes; statement of the results in absolute numbers when feasible		Sample characteristics + Figure 4
		• Indication of whether the analysis strategy was "intention to treat" or, if not, description of how non-compliers were treated in the analyses		Statistics
Outcomes and estimation	17	• For each primary and secondary outcome, a summary of results for each estimation study condition, and the estimated effect size and a confidence interval to indicate the precision		Primary and secondary

Table S4. TREND Statement Checklist

				outcomes
		• Inclusion of null and negative findings		Primary and secondary outcomes
		• Inclusion of results from testing pre-specified causal pathways through which the intervention was intended to operate, if any		Primary and secondary outcomes
Ancillary analyses	18	• Summary of other analyses performed, including subgroup or restricted analyses, indicating which are pre-specified or exploratory		Primary and secondary outcomes
Adverse events	19	• Summary of all important adverse events or unintended effects in each study condition (including summary measures, effect size estimates, and confidence intervals)		not reported
**DISCUSSION**				
Interpretation	20	• Interpretation of the results, taking into account study hypotheses, sources of potential bias, imprecision of measures, multiplicative analyses, and other limitations or weaknesses of the study		Discussion
		• Discussion of results taking into account the mechanism by which the intervention was intended to work (causal pathways) or alternative mechanisms or explanations		Discussion
		• Discussion of the success of and barriers to implementing the intervention, fidelity of implementation		Discussion
		• Discussion of research, programmatic, or policy implications		Discussion
Generalizability	21	• Generalizability (external validity) of the trial findings, taking into account the study population, the characteristics of the intervention, length of follow-up, incentives, compliance rates, specific sites/settings involved in the study, and other contextual issues		Discussion
Overall evidence	22	• General interpretation of the results in the context of current evidence and current theory		Discussion

From: Des Jarlais, D. C., Lyles, C., Crepaz, N., & the Trend Group (2004). Improving the reporting quality of nonrandomized evaluations of behavioral and public health interventions: The TREND statement. *American Journal of Public Health*, 94, 361-366. For more information, visit: http://www.cdc.gov/trendstatement/
